# A Curious Case of Severe Recurrent Hypocalcemia

**DOI:** 10.7759/cureus.68271

**Published:** 2024-08-31

**Authors:** Sarah Alam, Sarla Kumari, Mukesh Kumar, Emilia Kadour, Vyomesh Buch

**Affiliations:** 1 Endocrinology, Canadian Specialist Hospital, Dubai, ARE; 2 Endocrinology, All India Institute of Medical Sciences, New Delhi, IND; 3 Internal Medicine, Canadian Specialist Hospital, Dubai, ARE; 4 Nephrology, Canadian Specialist Hospital, Dubai, ARE; 5 Radiology, Canadian Specialist Hospital, Dubai, ARE

**Keywords:** hyperphosphatemia, vitamin d deficiency, parathyroid hormone, pseudohypoparathyroidism, hypocalcemia

## Abstract

Hypocalcemia, characterized by low blood calcium levels, can range from asymptomatic to life-threatening. Common causes include hypoparathyroidism and vitamin D deficiency (VDD). Pseudohypoparathyroidism is a rare metabolic disorder marked by resistance to parathyroid hormone (PTH). This report details a young female presenting with severe hypocalcemia, hyperphosphatemia, and elevated PTH levels. She also had an associated VDD, which complicated the clinical picture. Despite receiving intravenous calcium and oral supplementation, she required extended treatment and readmission. Genetic testing revealed a variant in the CACNA1S gene. Her condition eventually stabilized with a strict, adjusted treatment regimen. This case underscores the importance of a systematic diagnostic approach, prolonged intravenous calcium therapy, and close monitoring. Pseudohypoparathyroidism represents a rare cause of severe hypocalcemia, emphasizing the need for close monitoring and regular follow-up to achieve improved outcomes.

## Introduction

Hypocalcemia, characterized by low blood calcium levels, is a common clinical condition with varied presentations. Acute hypocalcemia may require hospitalization, whereas chronic cases can be asymptomatic. Common symptoms of hypocalcemia include carpopedal spasms, muscle cramps, tetany, circumoral numbness, cognitive disturbances, and psychiatric manifestations such as anxiety, depression, or emotional instability. Severe cases can present with laryngospasm, prolonged QT intervals, heart failure, and intractable seizures. Hypocalcemia can result from various underlying causes, with hypoparathyroidism and vitamin D deficiency (VDD) being among the most common [1].

In adults, VDD rarely causes severe hypocalcemia due to the regulatory role of parathyroid hormone (PTH); however, in rapidly growing infants and children, VDD can lead to severe hypocalcemia and even seizures because their higher calcium demands are unmet [2]. Pseudohypoparathyroidism (PHP), characterized by resistance to PTH, is a rare cause of hypocalcemia. In some cases, VDD and PHP can coexist, complicating the clinical presentation [2].

In cases of severe hypocalcemia, prolonged intravenous calcium therapy may be essential to restore enterocyte function and optimize oral calcium absorption [3]. A systematic approach is essential for correct diagnosis, while ongoing follow-up is critical for optimal clinical management. Adherence to prescribed medications is crucial to mitigate complications and prevent recurrence in patients with PHP. This case highlights the management approach to PHP, an uncommon yet clinically significant cause of severe hypocalcemia.

## Case presentation

A 26-year-old female came to the United Arab Emirates as a tourist and presented to the ENT clinic with complaints of difficulty swallowing and throat discomfort. During the initial evaluation, she was found to have severe hypocalcemia and was subsequently referred to the endocrinology department for further assessment and management. On detailed history taking, it was found that she had a six-month history of throat discomfort, carpopedal spasms, tingling sensations, and generalized body pains, which had worsened over the past two months. She reported regular menstrual cycles and had a history of three full-term normal deliveries with no obstetric complications. There was no significant family history. On examination, she had a blood pressure of 118/80 mmHg, a pulse rate of 90/min, a height of 156 cm, a weight of 87 kg, and a BMI of 35.7 kg/m². She had a rounded face but no features of Albright's hereditary osteodystrophy (AHO). During the physical examination, Trousseau and Chvostek's signs were found to be positive. Her systemic examination was unremarkable.

Initial investigations revealed severe hypocalcemia (serum calcium: 4.7 mg/dL, normal reference range (N): 8.4‑10.2 mg/dL), hyperphosphatemia (serum phosphate: 6.9 mg/dL, N: 2.7‑4.7 mg/dL), slightly low magnesium level (1.3 mg/dL, N: 1.7-2.3 mg/dL), high PTH levels (230 pg/mL, N: 15‑65 pg/mL), and VDD (25‑hydroxyvitamin D 13.7 ng/mL, N: 30‑100 ng/mL).

Her initial workup, including complete blood count and liver and kidney function tests, was unremarkable. She was admitted and received a calcium gluconate infusion at 100 mg/hour for five hours and an injection of cholecalciferol 300,000 IU intramuscularly. During the hospital stay, the patient received intravenous calcium and was closely monitored with six-hourly serum calcium levels, continuous cardiac monitoring, electrolyte levels, and regular assessment of vital signs. Treatment adjustments were made accordingly based on the calcium levels. Repeat calcium after the first infusion was still low at 6.1 mg/dL, necessitating another intravenous calcium gluconate infusion at the same rate and duration, which increased calcium to 6.4 mg/dL and phosphorus to 7.8 mg/dL. She was also given oral magnesium carbonate 300 mg twice daily (BID) and alfacalcidol 0.5 mcg thrice daily (TID). She received a total of 1000 mg of intravenous calcium gluconate during this hospital admission, which led to symptomatic improvement, including the reduction of cramps and spasms. However, since her calcium levels remained below 7 mg/dL, she was advised to stay in the hospital for further monitoring and treatment, but she sought discharge against medical advice the next day. She was discharged on oral medications, namely calcium carbonate 1000 mg TID, alfacalcidol 0.5 mcg TID, magnesium 300 mg BID, cholecalciferol 50,000 IU once a week, and sevelamer 800 mg BID.

Two days following her discharge, the patient returned to the clinic experiencing a recurrence of her initial symptoms. Upon examination, both Chvostek's and Trousseau's signs were found to be positive. Remarkably, despite being on oral calcium supplements, her serum calcium level once again reached a severely low level (serum calcium: 4.7 mg/dL, N: 8.4‑10.2 mg/dL), and she had hyperphosphatemia (serum phosphate: 7.5 mg/dL, N: 2.7‑4.7 mg/dL). She was advised of urgent hospital re-admission and restarted on intravenous calcium gluconate infusion at 100 mg/hour for five hours, raising calcium to 6.1 mg/dL and phosphorus to 7.2 mg/dL. This was followed by another infusion at the same rate, with the calcium level rising to 6.9 mg/dL, and continuous intravenous calcium was continued at 100 mg/hour, increasing calcium to 7.0 mg/dL and phosphorus decreasing to 5.9 mg/dL. Oral treatments continued with phosphorus binders and magnesium. The dose of alfacalcidol was given at 1 mcg TID. After stopping the intravenous calcium infusion, calcium levels again dropped to 5.6 mg/dL with worsening symptoms. Intravenous calcium infusion was recommenced, and she was advised to continue her hospital stay. Her 24-hour urine calcium showed hypocalciuria (Table 1).

**Table 1:** Biochemical profile of the patient PTH: parathyroid hormone, 25(OH)D: 25-hydroxyvitamin D, ft4: free thyroxine, fT3: free triiodothyronine, TSH: thyroid-stimulating hormone

Parameter	Visit 1 clinic	Hospital admission 1	Hospital admission 1	Visit 2 clinic	Hospital admission 2	Hospital admission 2	Hospital admission 2	Visit 3 clinic	Visit 4 clinic	Visit 5 clinic	Visit 6 clinic	Visit 7 clinic	Visit 8 clinic	Visit 9 clinic	Reference range
		Day 1	Day 2		Day 1	Day 1	Day 2	After 2 days of discharge	After 1 week of discharge	After 1 month (before leaving for home country)	After 6 months	After the resumption of treatment		After 2 weeks	
Serum calcium (mg/dL)	4.7	6.1	6.4	4.7	6.1	6.9	6.7	6.0	7.1	9.3	6.5	7.3	8.5	9.4	8.4-10.2
Serum phosphate (mg/dL)	6.9	6.9	7.8	7.5	7.2	6.4	6.6	6.2	6.5	5.2	4.8	4.5	4.0	5.0	2.7-4.7
Magnesium (mg/dL)	1.3	1.8	-	1.8	-	-	-	1.9	1.8	1.8	1.6	1.7	1.7	-	1.7-2.3
Alkaline phosphatase (IU/L)	260	-	-	-	-	228	-	-	-	-	-	-	-	-	80-240
Creatinine (mg/dL)	0.6	-	-	-	-	-	-	-	-	-	0.5	-	-	-	0.5-1.2
Albumin (g/dL)	4.1	-	-	-	-	-	-	-	-	-	4.1	-	-	-	3.5-5.0
PTH (pg/mL)	230	-	-	-	-	219	-	-	-	-	258.3	-	-	-	15-65
25(OH)D (ng/mL)	13.7	-	-	-	-	19.8	-	-	-	29.3	25.4	-	-	32.6	<10 deficient, 10-30 insufficient, 30-100 sufficient
Urinary calcium (mg/24 h)	-	5.5	-	-	-	-	-	-	-	-	-	-	-	-	20-275
Urinary creatinine (mg/24 h)	-	1035.3	-	-	-	-	-	-	-	-	-	-	-	-	720-1510
fT3 (pg/mL)	-	3.2	-	-	-	-	-	-	-	-	-	-	-	-	2-4.4
fT4 (ng/dL)	-	1.45	-	-	-	-	-	-	-	-	-	-	-	-	0.71-1.85
TSH (uIU/mL)	-	3.36	-	-	-	-	-	-	-	-	-	-	-	-	0.47-4.64

**Figure 1:**
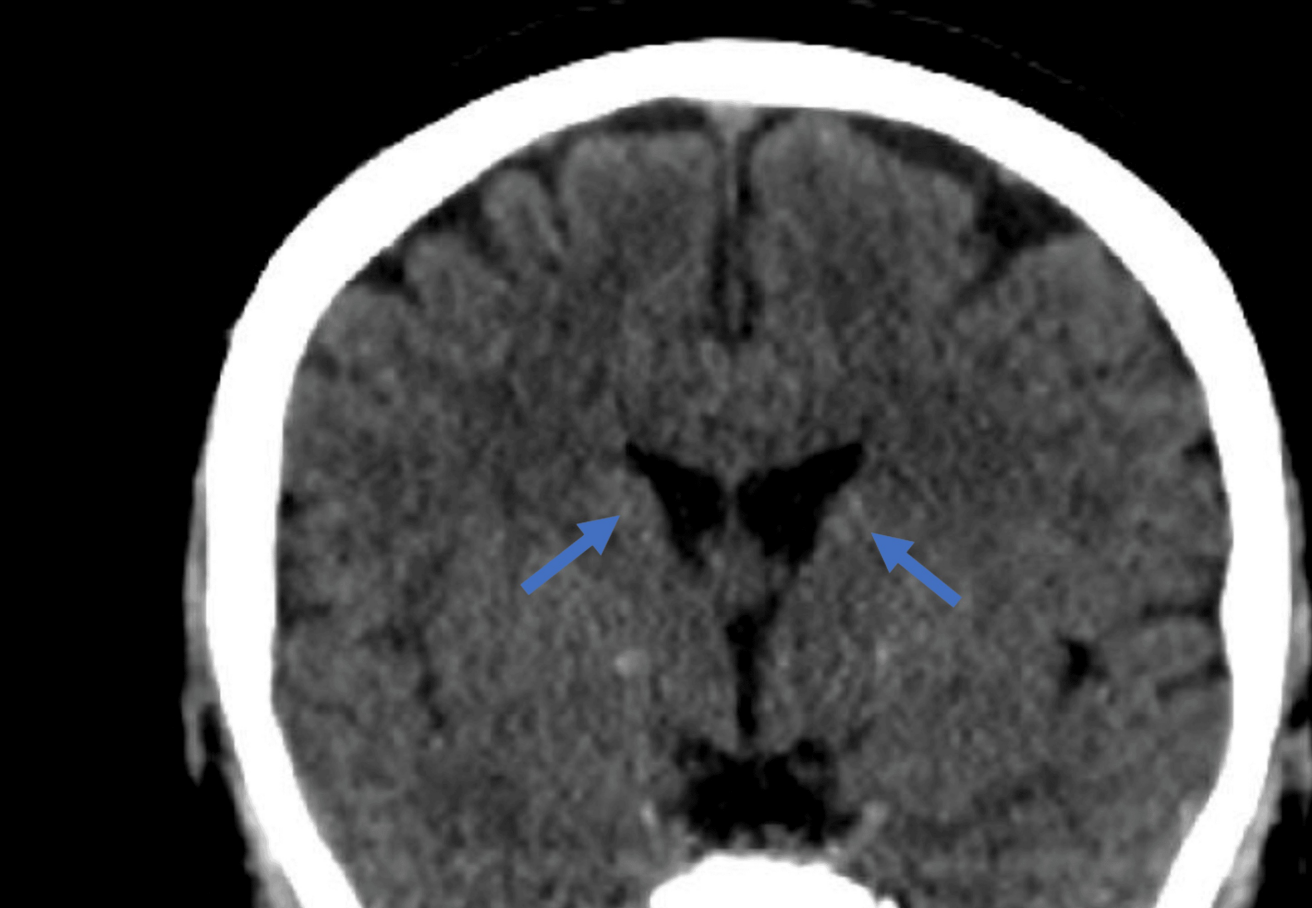
CT scan of the brain without contrast Bilateral basal ganglia (globus pallidus and head of the caudate nucleus) exhibit spotty calcifications (blue arrows).

Imaging, including USG abdomen and hand X-ray, was normal. There was no evidence of AHO. The CT brain showed bilateral basal ganglia calcifications but no other significant abnormalities (Figure 1). When serum calcium reached more than 7 mg/dL and she symptomatically improved along with optimization of her oral calcium dose, she was discharged from the hospital. To optimize her treatment, she was shifted from alfacalcidol to the more potent calcitriol, which is known to be twice as potent. On discharge, her regimen included calcium carbonate 1000 mg every four hours, calcitriol 1 mcg TID, magnesium 150 mg BID, cholecalciferol 50,000 IU weekly, and sevelamer 1600 mg TID. She had received a single IM dose of 300,000 IU cholecalciferol in the previous admission. Despite this, her repeat 25-hydroxyvitamin D levels remained low at 19.8 ng/mL. To address this, she was prescribed an additional course of oral cholecalciferol at 50,000 IU weekly for eight weeks to achieve adequate vitamin D levels and manage her hypocalcemia. For the evaluation of PHP, whole exome sequencing (WES) was sent.

One month later, her follow-up showed improved calcium (9.3 mg/dL) and phosphorus (5.2 mg/dL) levels, with 25-hydroxyvitamin D at 29.3 ng/mL. Her medication doses were reduced. There was an availability issue with calcitriol due to a local stock shortage, and she was shifted back to alfacaldiol. Her doses were reduced to calcium carbonate 1000 mg BID, alfacalcidol 1 mcg BID, and continued cholecalciferol 50,000 IU once a week. The WES using next-generation sequencing (NGS) technology of the Proband and NGS-based copy number variation analysis revealed a heterozygous missense variant (c.2245G>A, p.G1u749Lys) in the CACNA1S gene, associated with thyrotoxic periodic paralysis susceptibility to 1 (TTPP1). This variant is classified as a variant of uncertain significance. Her thyroid function test and thyroid ultrasound were normal. She then returned to her home country.

She adhered to her medication regimen for three months but subsequently discontinued all medications. Six months after her last visit, she returned to the clinic, having been off medications for three months. She reported experiencing tingling and numbness persisting for three months, with symptoms worsening over the past month.

Moreover, she revealed that she missed her menses and was found to be 12 weeks pregnant. On examination, a positive Chvostek's sign was noted. Her blood investigations revealed severe hypocalcemia (serum calcium: 6.5 mg/dL, N: 8.4‑10.2 mg/dL) and hyperphosphatemia (serum phosphate: 4.8 mg/dL, N: 2.7‑4.7 mg/dL). She also had high PTH levels (258.3 pg/mL, N: 15‑65 pg/mL), normal magnesium level (1.6 mg/dL, N: 1.7-2.3 mg/dL), and low vitamin D (25‑hydroxyvitamin D 25.4 ng/mL, N: 30‑100 ng/mL). She had severe hypocalcemia and was advised to be admitted to the hospital, but she refused. Therefore, she was started on oral calcium supplementation with calcium carbonate at 1,000 mg TID, alfacalcidol at 1 mcg TID, magnesium at 400 mg daily, and cholecalciferol at 50,000 IU weekly.

A close monitoring plan was implemented with dose adjustments as necessary. Subsequent follow-up revealed improvement, allowing for a reduction in calcium doses. After one week, her follow-up revealed improvement in calcium and phosphate levels and vitamin D levels. Symptomatically, she showed improvement and continues to fare well. At the 10-day follow-up, further enhancement in calcium levels was noted, leading to a reduction in dosage to calcium carbonate 1,000 mg BID, alfacalcidol 1 mcg BID, magnesium 400 mg daily, and cholecalciferol 2,000 IU daily.

## Discussion

Hypocalcemia is a frequently encountered biochemical abnormality, varying in severity from asymptomatic in mild cases to life-threatening situations. The main differential diagnoses of hypocalcemia with hyperphosphatemia and high PTH, with normal renal function, include PHP and VDD [1]. PHP is a rare disorder characterized by low serum calcium, elevated serum phosphate, and end-organ resistance to PTH leading to high PTH levels. It is generally diagnosed during childhood but can present later in life as well [4].

PHP is classified into Type 1 and Type 2 based on the urinary cAMP response following intravenous PTH injection. In Type 1 PHP, there is a lack of cAMP increase after PTH administration, while Type 2 PHP exhibits an increase in cAMP levels. Type 1 is further subdivided into 1a, 1b, or 1c, with subtypes 1a and 1c typically inheriting an autosomal dominant pattern and often presenting with the Albright osteodystrophy phenotype along with multi-hormone resistance [5].

Subtype 1b is characterized by resistance specific to PTH, often without other symptoms, and is usually sporadic. Type 2 PHP is characterized by the absence of a known molecular defect and does not present with an AHO phenotype or resistance to multiple hormones. Patients with PHP Type 2 may exhibit an acquired defect secondary to VDD, and replacement therapy with calcium and vitamin D has been even shown to normalize the phosphaturic response to PTH in these individuals [4]. Based on these findings, we diagnosed her with either Type 1b or Type 2 PHP, as she does not exhibit the Albright phenotype or resistance to multiple hormones. Patients with AHO typically exhibit short stature, a round face, obesity, brachydactyly, shortened metatarsals, reduced intelligence, and ectopic ossifications [6].

In cases of severe hypocalcemia, an extended duration of intravenous calcium is often necessary to stabilize serum calcium levels before transitioning to oral calcium supplementation, especially when predisposing risk factors impair effective oral calcium absorption [7]. Several factors can impair oral calcium absorption, including VDD, gastrointestinal disorders like Crohn’s disease, celiac disease, or post-gastric bypass surgery, hypomagnesemia, chronic kidney disease, and medications such as proton pump inhibitors (PPI) and glucocorticoids. In the case presented, the patient's VDD was a major risk factor, likely contributing to the impaired absorption of oral calcium, and she also had a history of PPI use [7]. Close monitoring and ongoing dosage adjustments are essential in managing such cases of hypocalcemia for stabilization and to prevent the recurrence of symptoms. Intravenous calcium should be continued until the patient transitions to an effective regimen of oral calcium and vitamin D. For optimal outcomes, it is beneficial to continue hospitalization until serum calcium levels are normalized [8].

Patients with PHP are functionally "hypoparathyroid," but due to a normal PTH response in bone, they can present with hyperparathyroid bone disease (osteitis fibrosa cystica) due to elevated PTH levels [4]. Concomitant VDD leads to higher PTH levels, as a normal physiological response to VDD is an increased secretion of PTH. Lowering PTH levels with appropriate doses of calcium and vitamin D typically reduces intact PTH levels [9]. Therefore, aggressive therapy is recommended to maintain lower PTH levels effectively. Moreover, unlike in hypoparathyroidism, the intact anti-calciuric action of PTH in the thick ascending limb is unaffected in PHP, leading to a reduced risk of hypercalciuria, even with aggressive calcium and vitamin D therapy [4]. However, specific assessments for hyperparathyroid bone disease, such as bone mineral density evaluation or measurement of bone microarchitecture through trabecular bone score, were not conducted in this case.

The WES was performed in this patient; however, the variant detected is of unknown significance. Re-evaluating WES data has the potential to increase diagnostic accuracy in patients who did not receive a definitive molecular diagnosis initially, and this should be considered [10]. Point mutations are effectively identified through sequencing methods like Sanger sequencing or NGS, with careful attention needed for full coverage of exon 1 due to its CG-rich nature. Genomic rearrangements are best detected using quantitative techniques such as multiplex ligation-dependent probe amplification (MLPA) or comparative genomic hybridization arrays (aCGH) [11]. Ideally, both NGS and MLPA should be utilized to detect point mutations and potential genomic rearrangements comprehensively. In our case, we used only NGS to identify point mutations, recognizing the importance of MLPA or aCGH for detecting larger genomic rearrangements. Unfortunately, financial and availability constraints prevented us from performing these additional tests.

The treatment goal is to achieve normal serum calcium levels and reduce PTH hypersecretion. To achieve this, vitamin D and calcium supplements were administered [4]. Through careful monitoring and adherence to treatment, the patient's condition improved significantly.

## Conclusions

The patient presented with a complex condition characterized by severe hypocalcemia necessitating prolonged intravenous calcium. This case underscores the need for extended hospitalization for high-dose intravenous calcium, particularly in patients with predisposing risk factors. Patients with severe hypocalcemia require close monitoring, follow-up, and consistent adherence to therapy. VDD may coexist in patients with PHP, complicating the clinical picture. If the biochemical picture of hypocalcemia, hyperphosphatemia, and elevated PTH persists after vitamin D repletion, PHP is the likely diagnosis.
